# Supplementary material for: Study of Helicobacter pylori Isolated from a High-Gastric-Cancer-Risk Population: Unveiling the Comprehensive Analysis of Virulence-Associated Genes including Secretion Systems, and Genome-Wide Association Study
Source: Cancers (Basel). 2023 Sep 12;15(18):4528. doi: 10.3390/cancers15184528 (PMC10526929; doi:10.3390/cancers15184528)
Supplement: Supplementary file 1 [file cancers-15-04528-s001.zip › cancers-2509055-Supplementary files.pdf]

Supplementary files

Study of *Helicobacter pylori* Isolated from a High-Gastric-Cancer-Risk Population: Unveiling the Comprehensive Analysis of Virulence-Associated Genes Including Secretion Systems, and Genome-wide Association Study

Batsaikhan Saruuljavkhlan [1], Ricky Indra Alfaray [1,2], Khasag Oyuntsetseg [3], Boldbaatar Gantuya [3,4], Ayush Khangai [1], Renchinsengee Namsrai [1], Takashi Matsumoto [1], Junko Akada [1], Dashdorj Azzaya [4], Duger Davaadorj [4], and Yoshio Yamaoka [1,2,5,6*]

[1] Department of Environmental and Preventive Medicine, Oita University Faculty of Medicine, Yufu, Oita 879-5593, Japan; saruuljavkhlan@yahoo.com (B.S.); rickyindraalfaray@gmail.com (R.I.A); m20d9103@oita-u.ac.jp (A.K.); m22d9105@oita-u.ac.jp (R.N.); tmatsumoto9@oita-u.ac.jp (T.M.); akadajk@oita-u.ac.jp (J.A.);

[2] *Helicobacter pylori* and Microbiota Study Group, Institute of Tropical Disease, Universitas Airlangga, Surabaya, East Java 60286, Indonesia

[3] Endoscopy Center, Mongolia Japan Hospital, Mongolian National University of Medical Sciences, Ulaanbaatar 14210, Mongolia; oyuntsetseg.kh@mnums.edu.mn (K.O.), gantuya@mnums.edu.mn (B.G.)

[4] Department of Gastroenterology and Hepatology, Mongolian National University of Medical Sciences, Ulaanbaatar 14210, Mongolia; azzaya2000@gmail.com (D.A.); davaadorj@mnums.edu.mn (D.D.);

[5] The Research Center for GLOBAL and LOCAL Infectious Diseases (RCGLID), Oita University, Yufu, Oita 870-1192, Japan

[6] Department of Medicine, Gastroenterology and Hepatology Section, Baylor College of Medicine, Houston, Texas 77030, USA

* Correspondence: yyamaoka@oita-u.ac.jp; Tel.: +81-97-586-5740; Fax: +81-97-586-5749

2023

Table S1. The gene list of HpVAG dataset

No	Gene locus by 26,695	Gene name	Gene function	Functional groups
**Virulence associated genes**				
1	HP0243	*nap*	Neutrophil-activating protein	Binding or transport proteins
2	HP0298	*ddpA*	Periplasmic dipeptide-binding protein	Binding or transport proteins
3	HP0508	*pgbA*	Plasminogen-binding protein A	Binding or transport proteins
4	HP0888/HP0889	*HP0888*	Iron (III) dicitrate ABC transporter, ATP-binding protein	Binding or transport proteins
6	HP0970	*nccB*	Nickel-cobalt-cadmium import protein B	Binding or transport proteins
7	HP1073	*copP*	Copper-binding protein P	Binding or transport proteins
8	HP1252	*oppA*	Oligopeptide ABC transporter periplasmic oligopeptide-binding protein A	Binding or transport proteins
9	HP1286	*HP1286*	YceI-like acidic stress response, lipocalin	Binding or transport proteins
10	HP1561/HP1562	*ceuE*	ABC transporter periplasmic binding protein E	Binding or transport proteins
12	HP0019	*cheV1*	Chemotaxis coupling protein V1	flagellar component
13	HP0115	*flaB*	Flagellin B	flagellar component
14	HP0173	*fliR*	Export component/flagellar biosynthetic protein R	flagellar component
15	HP0178[a]	*pseI*	Pseudaminic acid synthase	flagellar component
16	HP0244	*flgS*	Signal-transducing protein, histidine kinase	flagellar component
17	HP0246	*flgI*	P-ring protein; part of bushing; internal disulfide bridge	flagellar component
18	HP0256	*fliJ*	General chaperone	flagellar component
19	HP0295	*flgL*	Hook-associated protein 3; second hook-filament junction protein	flagellar component
20	HP0325	*flgH*	L-ring protein; part of bushing; lipoprotein	flagellar component
21	HP0326[a]	*pseFG*	CMP-N-acetylneuraminic acid synthetase	flagellar component
22	HP0327[a]	*flaG1/pseH/flmH*	Flagellar protein G homolog 1, Pseudaminic acid biosynthesis protein H	flagellar component
23	HP0351	*fliF*	MS-ring protein; mounting flange for rotor/ switch and rod; housing for export apparatus	flagellar component
24	HP0352	*fliG*	Rotor/switch protein; torque generation; strong interaction with MS ring	flagellar component
25	HP0353	*fliH*	Flagellar assembly protein H	flagellar component
26	HP0366	*pseC*	Polysaccharide biosynthesis protein C	flagellar component
27	HP0391	*cheW*	Chemotaxis coupling protein W	flagellar component
28	HP0392[a]	*cheA*	Chemotaxis protein A	flagellar component
29	HP0393	*cheV2*	Chemotaxis coupling protein V2	flagellar component
30	HP0492[a]	*HP_RS02435*	Neuraminyllactose-binding hemagglutinin	flagellar component
31	HP0518	*cds6*	L, D-carboxypeptidase	flagellar component
32	HP0584	*fliN*	Flagellar rotor/switch protein N	flagellar component
33	HP0599	*cheV3*	Chemotaxis coupling protein V3	flagellar component
34	HP0601	*flaA*	Flagellin A	flagellar component
35	HP0616[a]	*cheV2*	Chemotaxis protein V2	flagellar component
36	HP0685	*fliP*	Flagellar biosynthetic protein P	flagellar component
37	HP0703	*flgR*	Transcriptional activator of flagellar proteins R	flagellar component
38	HP0714[a]	*rpoN*	RNA polymerase factor sigma-54	flagellar component

39	HP0751	*flaG*	Flagellar protein G	flagellar component
40	HP0752	*fliD*	Flagellar hook-associated protein D	flagellar component
41	HP0753	*fliS*	Flagellar protein S	flagellar component
42	HP0770	*flhB*	Flagellar biosynthetic protein B	flagellar component
43	HP0809	*fliL*	Flagellar basal body protein L	flagellar component
44	HP0815	*motA*	Flagellar motor rotation protein A	flagellar component
45	HP0816	*motB*	Flagellar motor rotation protein B	flagellar component
46	HP0840	*flaA1*	FlaA1 protein	flagellar component
47	HP0870	*flgE_1*	Flagellar hook protein E1	flagellar component
48	HP0907	*flgD*	Hook-capping protein D	flagellar component
49	HP0908	*flgE*	Flagellar hook protein E	flagellar component
50	HP0958[a]	*flgZ*	Zinc ribbon domain-containing protein	flagellar component
51	HP1030	*fliY*	Flagellar motor switch protein Y	flagellar component
52	HP1031	*fliM*	Flagellar motor switch protein M (target for CheY-P binding)	flagellar component
53	HP1032	*fliA*	Flagellar biosynthesis sigma factor A	flagellar component
54	HP1034	*ylxH*	Flagellum site-determining protein H	flagellar component
55	HP1035	*flhF*	Flagellar biosynthesis protein	flagellar component
56	HP1041	*flhA*	Export component; target for soluble export complex	flagellar component
57	HP1067	*cheY*	Chemotaxis response regulator Y	flagellar component
58	HP1092	*flgG*	Flagellar basal-body rod protein G	flagellar component
59	HP1119	*flgK*	Flagellar hook-associated protein 1 K	flagellar component
60	HP1122	*flgM*	Negative regulator of flagellin synthesis, anti-sigma28 factor	flagellar component
61	HP1274	*pflA*	Paralysed flagella protein	flagellar component
62	HP1419	*fliQ*	Flagellar export component Q	flagellar component
63	HP1420	*fliI*	Flagellar export protein ATP synthase	flagellar component
64	HP1462[a]	*HP_RS07240*	META domain-containing protein	flagellar component
65	HP1477	*flgA*	Chaperone for P-ring protein Y	flagellar component
66	HP1557	*fliE*	Flagellar basal-body protein E	flagellar component
67	HP1558	*flgC*	Flagellar basal-body rod protein C; transmission shaft	flagellar component
68	HP1559	*flgB*	Flagellar basal-body rod protein B; transmission shaft	flagellar component
69	HP1575	*flhB2*	ABC transporter, homolog of FlhB protein	flagellar component
70	HP1582	*pdxJ*	Pyridoxal phosphate biosynthetic protein J	flagellar component
71	HP1583	*pdxA*	Pyridoxal phosphate biosynthetic protein A	flagellar component
72	HP1585[a]	*flgG_2*	Flagellar protein G homolog 2, Distal rod protein	flagellar component
73	HP0122	*HP0122*	Hypothetical uncharacterized protein	Hypothetical proteins
74	HP0129	*HP0129*	Hypothetical uncharacterized protein	Hypothetical proteins
75	HP0130	*HP0130*	Hypothetical uncharacterized protein	Hypothetical proteins
76	HP0135	*HP0135*	Hypothetical uncharacterized protein	Hypothetical proteins
77	HP0149	*HP0149*	Hypothetical uncharacterized protein	Hypothetical proteins
78	HP0169	*HP0169*	Hypothetical secreted collagenase	Hypothetical proteins
79	HP0204	*HP0204*	Hypothetical uncharacterized protein	Hypothetical proteins
80	HP0367	*HP0367*	Hypothetical uncharacterized protein	Hypothetical proteins
81	HP0555	*HP0555*	Hypothetical uncharacterized protein	Hypothetical proteins
82	HP0659	*HP0659*	Hypothetical uncharacterized protein	Hypothetical proteins
83	HP0719	*HP0719*	Hypothetical uncharacterized protein	Hypothetical proteins
84	HP0720[a]	*HP0720*	Hypothetical protein	Hypothetical proteins
85	HP0721	*HP0721*	Sialic acid-specific adhesion, metal homeostasis	Hypothetical proteins
86	HP0781	*HP0781*	Hypothetical uncharacterized protein	Hypothetical proteins
87	HP0783	*HP0783*	Hypothetical uncharacterized protein	Hypothetical proteins

88	HP0902	*HP0902*	Hypothetical uncharacterized protein	Hypothetical proteins
89	HP0953	*HP0953*	Hypothetical uncharacterized protein	Hypothetical proteins
90	HP0973	*HP0973*	Hypothetical uncharacterized protein	Hypothetical proteins
91	HP1023	*HP1023*	Hypothetical uncharacterized protein	Hypothetical proteins
92	HP1055	*HP1055*	Hypothetical uncharacterized protein	Hypothetical proteins
93	HP1056	*HP1056*	Hypothetical uncharacterized protein	Hypothetical proteins
94	HP1057	*HP1057*	Hypothetical uncharacterized protein	Hypothetical proteins
95	HP1173	*HP1173*	Hypothetical uncharacterized protein	Hypothetical proteins
96	HP1285	*HP1285*	Lipoprotein, e(P4) family	Hypothetical proteins
97	HP1454	*HP1454*	Hypothetical uncharacterized protein	Hypothetical proteins
98	HP1527	*HP1527*	Hypothetical uncharacterized protein	Hypothetical proteins
99	HP1580	*HP1580*	Hypothetical uncharacterized protein	Hypothetical proteins
100	HP0043	*rfbM*	Mannose-6-phosphate isomerase	Immune modulation
101	HP0044	*rfbD/gmd*	GDP-D-mannose dehydratase	Immune modulation
102	HP0045	*wbcJ*	GDP fucose synthase	Immune modulation
103	HP0159	*rfaJ*	Alpha-1,6 Glc transferase	Immune modulation
104	HP0279	*rfaC/waaC*	Lipopolysaccharide heptosyltransferase-1	Immune modulation
105	HP0360	*gluE/galE*	UDP-glucose 4-epimerase	Immune modulation
106	HP0679	*wbpB*	Lipopolysaccharide biosynthesis protein B	Immune modulation
107	HP0867	*lpxB*	Lipid A disaccharide synthetase B	Immune modulation
108	HP1174	*gluP*	Glucose/galactose transporter/sugar MFS transporter P	Immune modulation
109	HP1416[a]	*HP_RS07005*	Alpha-1,2/4 Glc transferase	Immune modulation
110	HP1475	*kdtB*	Lipopolysaccharide core biosynthesis protein	Immune modulation
111	HP0004	*icfA*	Carbonic anhydrase	Other enzymes
112	HP0010	*hspA*	Chaperone and heat shock protein, HspR-dependent regulon A	Other enzymes
113	HP0011	*hspB*	Cochaperone, HspR-dependent regulon B	Other enzymes
114	HP0026	*gltA*	Citrate synthase	Other enzymes
115	HP0154	*eno*	Enolase	Other enzymes
116	HP0166	*arsS*	Response regulator S	Other enzymes
117	HP0194	*tpiA*	Triosephosphate isomerase A	Other enzymes
118	HP0275	*addB*	ATP-dependent nuclease B	Other enzymes
119	HP0294	*aimE*	Aliphatic amidase E	Other enzymes
120	HP0305	*HP0305*	Putative human regulator of G protein signaling 12	Other enzymes
121	HP0310	*HP0310*	Polysaccharide deacetylase	Other enzymes
122	HP0323	*nuc*	Membrane-bound endonuclease	Other enzymes
123	HP0380	*HP0380*	Glutamate dehydrogenase	Other enzymes
124	HP0672	*aspB*	Aspartate aminotransferase B	Other enzymes
125	HP0743	*HP0743*	Rod shape-determining protein	Other enzymes
126	HP0827	*HP0827*	ssDNA-binding 12RNP2 precursor	Other enzymes
127	HP0835	*HP0835*	Histone-like DNA-binding protein HU	Other enzymes
128	HP1000	*HP1000*	PARA protein	Other enzymes
129	HP1118	*HP1118*	γ-Glutamyltranspetptidase	Other enzymes
130	HP1126	*tolB*	Colicin tolerant protein B	Other enzymes
131	HP1178	*HP1178*	Purine nucleoside phosphorylase DeoD-type	Other enzymes
132	HP1186	*HP1186*	Carbonic anhydrase	Other enzymes
133	HP1202	*HP1202*	Ribosomal protein L11	Other enzymes
134	HP1375	*lpxA*	UDP-N-acetylglucosamine acyltransferase	Other enzymes
135	HP1432	*hpn2*	Hpn-like protein (Nickel-binding protein L)	Other enzymes
136	HP0175	*surA*	Secreted peptide-based proline cis-trans isomerase	Outer membrane biogenesis complex components
137	HP0320	*tatA*	Sec-independent protein translocase protein A	Outer membrane biogenesis complex components

138	HP0362	*lptF*	Integral membrane protein F	Outer membrane biogenesis complex components
139	HP0378	*bamD*	Outer membrane-associated lipoprotein D	Outer membrane biogenesis complex components
140	HP0655	*bamA*	Outer membrane β-barrel assembly component A	Outer membrane biogenesis complex components
141	HP0715	*lptB*	Outer membranes lipopolysaccharide transport protein Inner membrane ATP-binding-cassette transporter domain	Outer membrane biogenesis complex components
142	HP0763	*ftsY*	SRP receptor	Outer membrane biogenesis complex components
143	HP0785	*lolA*	Periplasmic chaperone A	Outer membrane biogenesis complex components
144	HP0786	*secA*	Preprotein translocase subunit A	Outer membrane biogenesis complex components
145	HP0787	*lolC (lolE)*	Integral membrane protein E	Outer membrane biogenesis complex components
146	HP1060	*tatB*	Sec-independent translocase B	Outer membrane biogenesis complex components
147	HP1061	*tatC*	Sec-independent translocase C	Outer membrane biogenesis complex components
148	HP1082	*msbA*	Inner membrane LPS flippase	Outer membrane biogenesis complex components
149	HP1152	*ffh*	Signal recognition particle (SRP)	Outer membrane biogenesis complex components
150	HP1203	*secE*	Preprotein translocase subunit E	Outer membrane biogenesis complex components
151	HP1216	*lptD*	Outer membranes lipopolysaccharide transport protein D	Outer membrane biogenesis complex components
152	HP1255	*secG*	Preprotein translocase subunit	Outer membrane biogenesis complex components
153	HP1300	*secY*	Preprotein translocase subunit	Outer membrane biogenesis complex components
155	HP1498	*lptG*	Outer membranes lipopolysaccharide transport protein G, integral membrane protein	Outer membrane biogenesis complex components
156	HP1540	*yidC*	Inner membrane protein translocase component C	Outer membrane biogenesis complex components
157	HP1546	*lptE*	Outer membranes lipopolysaccharide transport protein E	Outer membrane biogenesis complex components
158	HP1549	*secF*	Preprotein translocase subunit F	Outer membrane biogenesis complex components
159	HP1550	*secD*	Preprotein translocase subunit D	Outer membrane biogenesis complex components
160	HP1551	*yajC*	Preprotein translocase subunit C	Outer membrane biogenesis complex components
161	HP1568	*lptA*	Outer membranes lipopolysaccharide transport protein A, Periplasmic chaperone	Outer membrane biogenesis complex components
162	HP1569	*lptC*	Outer membranes lipopolysaccharide transport protein C	Outer membrane biogenesis complex components
163	HP0570	*pepA*	Aminopeptidase A	Proteases
164	HP0657	*ymxG*	Processing protease	Proteases
165	HP1012	*HP1012*	Putative Zinc protease	Proteases
166	HP1018/HP1019	*htrA*	Secreted serine protease and chaperone high temperature requirement A	Proteases
167	HP1037	*HP1037*	Amino peptidase	Proteases
168	HP1350	*HP1350*	Carboxyl-terminal protease	Proteases
169	HP0160	*hcpD*	*H. pylori* cysteine-rich protein D	Putative solenoid proteins

170	HP0211	*hcpA*	*H. pylori* cysteine-rich protein A	Putative solenoid proteins
171	HP0235	*hcpE*	*H. pylori* cysteine-rich protein E	Putative solenoid proteins
172	HP0336	*hcpB*	*H. pylori* cysteine-rich protein B	Putative solenoid proteins
173	HP0519	*HP0519*	Hypothetical Sel1-like protein	Putative solenoid proteins
174	HP0628	*HP0628*	Hypothetical Sel1-like protein	Putative solenoid proteins
175	HP1098	*hcpC*	*H. pylori* cysteine-rich protein C	Putative solenoid proteins
176	HP1117	*hcpX*	*H. pylori* cysteine-rich protein X	Putative solenoid proteins
177	HP1124	*HP1124*	Tetratricopeptide-like repeat protein	Putative solenoid proteins
178	HP0224	*msrA/B*	Peptide methionine sulfoxide reductase A/B	Redox systems and the electron transport chain
179	HP0231	*dsbG*	Disulphide interchange protein G	Redox systems and the electron transport chain
180	HP0330	*ilvC*	Ketol-acid reductoisomerase	Redox systems and the electron transport chain
181	HP0377	*dsbC*	Disulphide interchange protein C	Redox systems and the electron transport chain
182	HP0389	*sod*	Superoxide dismutase	Redox systems and the electron transport chain
183	HP0390	*tagD*	Adhesin-thiol peroxidase D	Redox systems and the electron transport chain
184	HP0485	*HP0485*	Catalase-like protein	Redox systems and the electron transport chain
185	HP0824	*trxA* or *trx1*	Thioredoxin A	Redox systems and the electron transport chain
186	HP0825	*trxB* or *trx2*	Thioredoxin reductase	Redox systems and the electron transport chain
187	HP0875	*katA*	Catalase	Redox systems and the electron transport chain
188	HP1136	*HP1136*	ATP synthase subunit b	Redox systems and the electron transport chain
189	HP1161	*fldA*	Flavodoxin	Redox systems and the electron transport chain
190	HP1212	*HP1212*	ATP synthase subunit c	Redox systems and the electron transport chain
191	HP1227	*HP1227*	Cytochrome c-553	Redox systems and the electron transport chain
192	HP1266	*HP1266*	NADH-ubiquinone oxidoreductase, NQO3 subunit	Redox systems and the electron transport chain
193	HP1458	*trxC*	Thioredoxin C	Redox systems and the electron transport chain
194	HP1461	*HP1461*	Cytochrome c551 peroxidase	Redox systems and the electron transport chain
195	HP0067	*ureH*	Urease accessory protein H	Stress survival
197	HP0068	*ureG*	Urease accessory protein G	Stress survival
198	HP0069	*ureF*	Urease accessory protein F	Stress survival
199	HP0070	*ureE*	Urease accessory protein E, metallochaperone	Stress survival
200	HP0071	*ureI*	Acid-activated urea channel protein	Stress survival
201	HP0072	*ureB*	Urease beta subunit B, urea amidohydrolase	Stress survival
202	HP0073	*ureA*	Urease alpha subunit A	Stress survival
203	HP0082	*tlpC*	Transmembrane chemoreceptor C	Stress survival
204	HP0099	*tlpA*	Transmembrane chemoreceptor A	Stress survival
205	HP0103	*tlpB*	Transmembrane chemoreceptor B	Stress survival
206	HP0289	*imaA*	Immunomodulatory autotransporter A, vacA-like protein	VacA and other toxins
207	HP0596	*tip-α*	Tumor necrosis factor α-inducing protein	VacA and other toxins
208	HP0609/HP0610	*faaA*	Flagella-associated autotransporter A, VacA-like protein	VacA and other toxins

209	HP0887	*vacA*	Vacuolating cytotoxin A	VacA and other toxins
210	HP0922	*vlpC*	VacA-like protein C	VacA and other toxins
**Outer Membrane Proteins (OMPs) [27]**				
1	HP0009	*hopZ*	*H. pylori* outer membrane porin (OMP) Z – Omp1	Hop family
2	HP0025	*hopD*	*H. pylori* outer membrane porin D / 2	Hop family
3	HP0227	*hopM*	*H. pylori* outer membrane porin M / Omp5	Hop family
4	HP0229	*hopA*	*H. pylori* outer membrane porin A / Omp6	Hop family
5	HP0252	*hopF*	*H. pylori* outer membrane porin F / Omp7	Hop family
6	HP0253/0254	*hopG*	*H. pylori* outer membrane porin G / Omp8	Hop family
7	HP0477	*hopJ*	*H. pylori* outer membrane porin J / Omp12	Hop family
8	HP0638	*hopH*	*H. pylori* outer membrane porin H / Outer inflammatory protein (OipA) / Omp13	Hop family
9	HP0706	*hopE*	*H. pylori* outer membrane porin E / Omp15	Hop family
10	HP0722	*hopO*	*H. pylori* outer membrane porin O / Sialic acid binding adhesion B (SabB) / Omp16	Hop family
11	HP0725	*hopP*	*H. pylori* outer membrane porin P / Sialic acid binding adhesion A (SabA) / Omp17	Hop family
12	HP1243	*babA (hopS)*	*H. pylori* outer membrane porin S / Blood group antigen–binding adhesin A (BabA) / Omp28	Hop family
13	HP0912	*hopC*	*H. pylori* outer membrane porin C / Adherence-associated lipoprotein A (AlpA) / Omp20	Hop family
14	HP0913	*hopB*	*H. pylori* outer membrane porin B / Adherence-associated lipoprotein B (AlpB) / Omp21	Hop family
15	HP0923	*hopK*	*H. pylori* outer membrane porin K / Omp22	Hop family
16	HP1156	*hopI*	*H. pylori* outer membrane porin I / Omp25	Hop family
17	HP1157	*hopL*	*H. pylori* outer membrane porin L / Omp26	Hop family
18	HP0896	*babB (hopT)*	*H. pylori* outer membrane porin T / blood group antigen–binding adhesin B (BabB) / Omp19	Hop family
19	HP1177	*hopQ*	*H. pylori* outer membrane porin Q / Omp27	Hop family
20	HP1342	*hopN*	*H. pylori* outer membrane porin N / Omp29	Hop family
21	HP0317	*hopU*	*H. pylori* outer membrane porin U / Omp9	Hop family
22	HP0078/0079	*horA*	*hop* related protein A / Omp3	Hor proteins
23	HP0127	*horB*	*hop* related protein B / Omp4	Hor proteins
24	HP0324	*horC*	*hop* related protein C / Omp10	Hor proteins
25	HP1066	*horD*	*hop* related protein D	Hor proteins
26	HP0472	*horE*	*hop* related protein E / Omp11	Hor proteins
27	HP0671	*horF*	*hop* related protein F / Omp14	Hor proteins
28	HP0796	*horG*	*hop* related protein G	Hor proteins
29	HP1107	*horH*	*hop* related protein H / Omp23	Hor proteins
30	HP1113	*horI*	*hop* related protein I	Hor proteins
31	HP1469	*horJ*	*hop* related protein J / Omp31	Hor proteins
32	HP1501	*horK*	*hop* related protein K	Hor proteins
33	HP1395	*horL*	*hop* related protein L / Omp30	Hor proteins
34	HP0209	*hofA*	*Helicobacter* OMP family protein A	Hof family
35	HP1083	*hofB*	*Helicobacter* OMP family protein B	Hof family
36	HP0486	*hofC*	*Helicobacter* OMP family protein C	Hof family
37	HP0487	*hofD*	*Helicobacter* OMP family protein D	Hof family
38	HP0782	*hofE*	*Helicobacter* OMP family protein E	Hof family
39	HP0788	*hofF*	*Helicobacter* OMP family protein F	Hof family
40	HP0914	*hofG*	*Helicobacter* OMP family protein G	Hof family
41	HP1167	*hofH*	*Helicobacter* OMP family protein H	Hof family
42	HP0710	*homA*	*Helicobacter* outer membrane protein A	Hom family

43	NA(JHP0870)	*homB*	*Helicobacter* outer membrane protein B	Hom family
44	HP0373	*homC*	*Helicobacter* outer membrane protein C	Hom family
45	HP1453	*homD*	*Helicobacter* outer membrane protein D	Hom family
46	HP0686	*fecA-1*	FecA-like protein A-1	iron-regulated OMPs
47	HP0807	*fecA-2*	FecA-like protein A-2	iron-regulated OMPs
48	HP1400	*fecA-3*	FecA-like protein A-3	iron-regulated OMPs
49	HP0876	*frpB-1*	FrpB-like protein 1	iron-regulated OMPs
50	HP0915/0916	*frpB-2*	FrpB-like protein 2	iron-regulated OMPs
51	HP1512	*frpB-3*	FrpB-like protein 3	iron-regulated OMPs
52	HP0605	*hefA*	Efflux pump outer membrane protein A	efflux pump OMPs
53	HP0971	*hefD*	Efflux pump outer membrane protein D	efflux pump OMPs
54	HP1327	*hefG*	Efflux pump outer membrane protein G	efflux pump OMPs
55	HP0506	*csd3*	Caseinase	Other OMPs
56	HP0655	*bamA*	Outer membrane β-barrel assembly component	Other OMPs
57	HP0694	*lpxR*	Putative outer membrane protein	Other OMPs
58	HP0726	-	Putative outer membrane protein	Other OMPs
59	HP0358	-	Putative outer membrane protein	Other OMPs
60	HP1467	-	Putative outer membrane protein	Other OMPs
61	HP0325	*flgH*	Flagellar basal-body L-ring protein H	Other OMPs
62	HP0839		Putative outer membrane protein	Other OMPs
63	HP1125	*palA*	Putative outer membrane protein	Other OMPs
64	HP1456	*lpp20*	Putative outer membrane protein	Other OMPs

[a] – gene that did not detected at all in *H. pylori*-Mon strains

[b]NA – not applicable, we used the gene from strain J99.

Table S3. *H. pylori* reference strains list

No	Strain name	Isolated country	Isolated place	Subpopulations	Reference
1	SouthAfrica50	South Africa	Soweto, Bantu origin	hpAfrica2	Duncan et al. 2013
2	SouthAfrica20	South Africa	Soweto, Bantu origin	hpAfrica2	Duncan et al. 2013
3	SouthAfrica7	South Africa	Soweto, Bantu origin	hpAfrica2	Duncan et al. 2013
4	SA166A	South Africa	Ogies, Mpumalanga	hpAfrica2	Didelot et al. 2013
5	SA303C	South Africa	Ogies, Mpumalanga	hpAfrica2	Didelot et al. 2013
6	SA35A	South Africa	Ogies, Mpumalanga	hspAfrica1SAfrica	Didelot et al. 2013
7	SA158A	South Africa	Ogies, Mpumalanga	hspAfrica1SAfrica	Didelot et al. 2013
8	SA45A	South Africa	Ogies, Mpumalanga	hspAfrica1SAfrica	Didelot et al. 2013
9	SA46C	South Africa	Ogies, Mpumalanga	hspAfrica1SAfrica	Didelot et al. 2013
10	SA146A	South Africa	Ogies, Mpumalanga	hspAfrica1SAfrica	Didelot et al. 2013
11	GAMchjs114i	Gambia	n/a	hspAfrica1WAfrica	Human Microbiome Project
12	GAM231Ai	Gambia	n/a	hspAfrica1WAfrica	Human Microbiome Project
13	GAM260Bi	Gambia	n/a	hspAfrica1WAfrica	Human Microbiome Project
14	GAMchjs124i	Gambia	n/a	hspAfrica1WAfrica	Human Microbiome Project
15	GAMchjs117Ai	Gambia	n/a	hspAfrica1WAfrica	Human Microbiome Project
16	26695_Tomb	UK	n/a	hspNEurope	Tomb et al. 1997
17	SSR4	UK	Dublin, Ireland	hspNEurope	Kaisa Thorell et al 2017
18	SSR13	UK	Dublin, Ireland	hspNEurope	Kaisa Thorell et al 2017
19	SSR8	UK	Dublin, Ireland	hspNEurope	Kaisa Thorell et al 2017
20	HPAG1	Sweden	Kalix, Norrland	hspNEurope	Oh et al. 2006
21	Keto01	Russia	Sulomai	hspUral	HEL_AA7957AA
22	Keto65	Russia	Sulomai	hspUral	HEL_AA8054AA
23	Keto23	Russia	Sulomai	hspUral	HEL_AA8174AA
24	Khanty39	Russia	Muzhi, Shurishkari	hspUral	HEL_AA7342AA
25	Nentsy32	Russia	Novi-port	hspUral	HEL_AA2429AA
26	NAB47	India	n/a	hspIndia	Kaisa Thorell et al 2017
27	NAK7	India	n/a	hspIndia	Kaisa Thorell et al 2017
28	SNT49	India	Santal tribe	hspIndia	Kaisa Thorell et al 2017
29	India7	India	n/a	hspIndia	Kaisa Thorell et al 2017
30	Myanmar51	Myanmar	n/a	hspIndia	Kaisa Thorell et al 2017
31	HP15050_WA	Iran	n/a	hspSahul	Kaisa Thorell et al 2017
32	ausabrJ05	Australia	Aboriginal	hspSahul	Kaisa Thorell et al 2017
33	HP15060	Afghanistan	n/a	hspSahul	Kaisa Thorell et al 2017
34	HP15035_WA	Iran	n/a	hspSahul	Kaisa Thorell et al 2017
35	Neuguinea56	Papua New Guinea	Highlander	hspSahul	Kaisa Thorell et al 2017
36	Arg-53RA1	Argentina	Buenos Aires City	hspSWEurope	PRJNA601302
37	CL-MASP	Chile	n/a	hspSWEurope	PRJNA601302
38	Pt-8376	Portugal	Porto	hspSWEurope	PRJNA601302

39	Pt-7757	Portugal	Sta Maria Feira	hspSWEurope	PRJNA601302
40	VE-HV076	Venezuela	Caracas	hspSWEurope	PRJNA601302
41	Todzha110	Russia	Todzha	hspAltai	HEL_AA7233AA
42	Todzha114	Russia	Todzha	hspAltai	HEL_AA7967AA
43	Todzha120	Russia	Todzha	hspAltai	HEL_AA8146AA
44	TUVAB11	Russia	Kyzyl	hspAltai	HEL_AA7241AA
45	TUVAB24	Russia	Kyzyl	hspAltai	HEL_AA7521AA
46	CAN-005	Canada	Northwest Territories	hspIndigenousNAmerica	PRJNA601302
47	CAN-011	Canada	Northwest Territories	hspIndigenousNAmerica	PRJNA601302
48	CAN-095	Canada	Northwest Territories	hspIndigenousNAmerica	PRJNA601302
49	CAN-258	Canada	Northwest Territories	hspIndigenousNAmerica	PRJNA601302
50	CAN-136	Canada	Northwest Territories	hspIndigenousNAmerica	PRJNA601302
51	Puno120	Peru	Puno	hspIndigenousSAmerica	Kaisa Thorell et al 2017
52	Shi417	Peru	Shimaa village	hspIndigenousSAmerica	Kaisa Thorell et al 2017
53	Shi112	Peru	Shimaa village	hspIndigenousSAmerica	Kaisa Thorell et al 2017
54	Shi169	Peru	Shimaa village	hspIndigenousSAmerica	Kaisa Thorell et al 2017
55	Cuz20	Peru	Cuzco	hspIndigenousSAmerica	Kaisa Thorell et al 2017
56	51	South Korea	n/a	hpEastAsia	Kaisa Thorell et al 2017
57	HLJHP256	China	n/a	hpEastAsia	Kaisa Thorell et al 2017
58	UM038	Malaysia	n/a	hpEastAsia	Rehvathy et al. 2013
59	HLJHP271	China	n/a	hpEastAsia	Kaisa Thorell et al 2017
60	Yangon222	Myanmar	n/a	hpEastAsia	Kaisa Thorell et al 2017
61	SU2	Sudan	n/a	HpNEAfrica	HEL_AA7286AA
62	NIGH2494	Nigeria	n/a	HpNEAfrica	HEL_AA1182AA
63	ETH04	Ethiopia	n/a	HpNEAfrica	HEL_AA2420AA
64	ETH09	Ethiopia	n/a	HpNEAfrica	HEL_AA2422AA
65	Somalia3	Somalia	n/a	HpNEAfrica	Duncan et al. 2013

Table S4. General characteristics of study patients

Variables	Total of 223 patients
**Gender**	
Female	150 (70.9%)
Male	65 (29.1%)
**Mean age, years ± SE**	45.2 ± 14.0
**Age groups**	
18-29 years	34 (15.2%)
30-49 years	102 (45.7%)
50-69 years	75 (33.6%)
>69 years	12 (5.4%)
**Sampling place**	
Ulaanbaatar	83 (37.2%)
Khentii province	45 (20.2%)
Umnugovi province	37 (16.6%)
Khuvsgul province	30 (13.5%)
Uvs province	28 (12.6%)
**Disaese status**	
Non-atrophic gastritis (NAG)	120 (53.8%)
Peptic ulcer disease (PUD)	9 (4.0%)
Atrophic gastritis/Intestinal metaplasia (AG/IM)	78 (35.0%)
Gastric cancer (GC)	16 (7.2%)

Figure S1. Diverse virulence factor genes by disease status (A) and *H. pylori* subgroups (B). The *p* values were used to analyze the differences in the percentage of strains positive for the considered genes between disease status or two subgroups by Chi-Square test or Fisher's exact test.

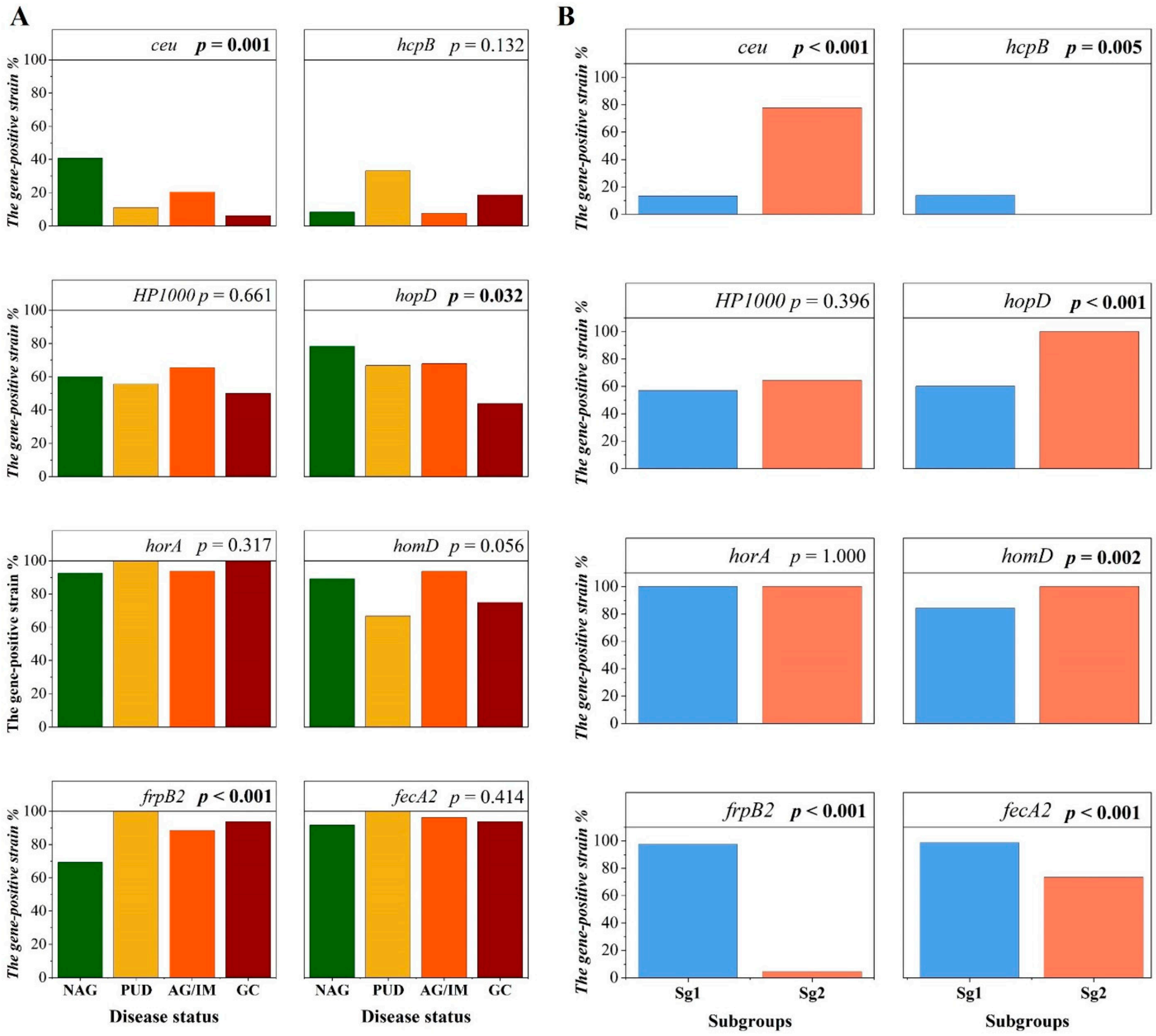

Table S5. Detected the allele types of *vacA* found in *H. pylori*-Mon

Simple s-i-m	Genotypes	Number	Percentages
s1-i1-m1	s1i1d1m1c1n1	98	43.9%
	s1i1d1m1c1n2	2	0.9%
	s1i1d1m1c2n1	3	1.3%
	s1i1d1m1c2n2	14	6.3%
	s1i1d2m1c1n1	5	2.2%
	s1i1d2m1c2n2	2	0.9%
	**subtotal**	**124**	**55.6%**
s1-i1-m2	s1i1d1m2c2n1	1	0.4%
	s1i1d1m2c2n2	12	5.4%
	s1i1d2m2c2n2	5	2.2%
	**subtotal**	**18**	**8.1%**

s1-i3-m2	s1i3d1m2c2n2	2	0.9%
	s1i3d2m2c1n1	1	0.4%
	s1i3d2m2c2n1	1	0.4%
	s1i3d2m2c2n2	27	12.1%
	**subtotal**	**31**	**13.9%**
s2-i3-m2	s2i3d1m2c2n2	1	0.4%
	s2i3d2m2c2n1	3	1.3%
	s2i3d2m2c2n2	35	15.7%
	**subtotal**	**39**	**17.5%**
other	s1i2d2m1c1n1	1	0.4%
	s1i3d1m1c2n2	1	0.4%
	s1i3d2m1c2n2	1	0.4%
	s2i2d2m2c1n1	1	0.4%
	s2i2d2m2c2n2	5	2.2%
	s2i3d2m1c2n2	2	0.9%
	**subtotal**	**11**	**4.9%**
	**Total**	**223**	**100%**

Table S6. Genotyping of selected OMP and virulence factor genes and *H. pylori* subgroups.

**Genes**	**Genotypes**	**Sg1 (*n*=158)**	**Sg2 (*n*=45)**	**Total (*n*=203)**	***P*[a]**
cagA	AB[1]	2 (1.3%)	0	2 (1.0%)	< 0.001
	ABC[2b]	92 (58.2%)	0	92 (45.3%)	
	ABCn[3b]	54 (34.2%)	0	54 (26.6%)	
	ABD[4]	4 (2.5%)	0	4 (2.0%)	
	Negative[b]	6 (3.8%)	45 (100%)	51 (25.1%)	
vacA	s1i1m1[b]	118 (74.7%)	0	118 (58.1%)	< 0.001
	s1i1m2[b]	17 (10.8%)	0	17 (8.4%)	
	s1i3m2	18 (11.4%)	3 (6.7%)	21 (10.3%)	
	s2i3m2[b]	1 (0.5%)	37 (82.2%)	38 (18.7%)	
	other[b]	4 (2.5%)	5 (11.1%)	9 (4.4%)	
htrA	S variant[b]	151 (95.6%)	20 (44.4%)	171 (84.2%)	< 0.001
	L variant[b]	7 (4.4%)	25 (55.6%)	32 (15.8%)	
iceA	Type 1[b]	84 (53.2%)	7 (15.6%)	91 (44.8%)	< 0.001
	Type 2A	6 (3.8%)	2 (4.4%)	8 (3.9%)	
	Type 2B[b]	9 (5.7%)	14 (31.1%)	23 (11.3%)	
	Type 2C[b]	13 (11.5%)	17 (37.8%)	30 (14.8%)	
	Type 2D[b]	46 (29.1%)	4 (8.9%)	50 (24.6%)	
	Negative	0	1 (2.2%)	1 (0.5%)	
babA	AD2	2 (1.3%)	1 (2.2%)	3 (1.5%)	0.377
	AD3	54 (34.2%)	10 (22.2%)	64 (31.5%)	
	AD4	99 (62.7%)	32 (71.1%)	131 (64.5%)	
	Negative	3 (1.9%)	2 (4.4%)	5 (2.5%)	
babB	BD1[b]	46 (29.1%)	2 (4.4%)	48 (23.6%)	< 0.001
	BD2	67 (42.4%)	12 (26.7%)	79 (38.9%)	
	BD3	24 (15.2%)	4 (8.9%)	28 (13.8%)	
	unclassified[b]	17 (10.8%)	11 (24.4%)	28 (13.8%)	

	Negative[b]	4 (2.5%)	16 (35.6%)	20 (9.9%)	
hopQ	Type I[b]	147 (93.0%)	0	147 (72.4%)	< 0.001
	Type II[b]	5 (3.2%)	45 (100%)	50 (24.6%)	
	Type I/II	6 (3.8%)	0	6 (3.0%)	
hopZ	Type I	150 (94.9%)	24 (55.8%)	174 (85.7%)	0.121
	Type II	8 (5.1%)	21 (46.7%)	29 (14.3%)	
sabA	Present	130 (82.3%)	45 (100%)	175 (86.2%)	< 0.001
	Negative	28 (17.7%)	0	28 (13.8%)	
sabB	Present	131 (82.9%)	0	131 (64.5%)	< 0.001
	Negative	27 (17.1%)	45 (100%)	72 (35.5%)	
homA/B	*homA*[b]	57 (36.1%)	36 (80.0%)	93 (45.8%)	< 0.001
	homB[b]	90 (57.0%)	8 (17.8%)	98 (48.3%)	
	homA/B	7 (4.4%)	0	7 (3.4%)	
	homI	4 (2.5%)	1 (2.2%)	5 (2.5%)	

[1]AB (AB n = 1, ABBB n = 1). [2]ABC (ABC n = 90, ABBBC n = 1, AABC n = 1). [3]ABCn (ABCC n = 51, ABCCC n = 3). [4]ABD (ABD n = 2, ABBD n = 1, ABDC n = 1). [a]Global p value between subgroups was assessed by Chi-Square test. [b]Adjusted $p < 0.05$ versus NAG group was determined by Fisher's exact test (Bonferroni method). Sg1 = subgroup 1, Sg2 = subgroup 2.

Table S7. Determination of the functional status of OMP genes and their association with disease groups and *H. pylori* subgroups.

Genes	Status	NAG *n* (%)	PUD *n* (%)	AG/IM *n* (%)	GC *n* (%)	Total *n* (%)	P[a]	Sg1 *n* (%)	Sg2 *n* (%)	Total *n* (%)	P[a]
oipA	On	82 (68.3)	9 (100)[b]	69 (88.5)[b]	16 (100)[b]	176 (78.9)	< 0.001	153 (96.8)	4 (8.9)	157 (77.3)	< 0.001
	Off	38 (31.7)	0 (0)[b]	9 (11.5)[b]	0 (0)[b]	47 (21.1)		5 (3.2)	41 (91.1)	46 (22.7)	
babB	On	63 (52.5)	4 (44.4)	43 (55.1)	11 (68.8)	121 (54.3)	0.305	98 (62.0)	9 (20.0)[b]	107 (52.7)	< 0.001
	Off	44 (36.7)	5 (55.6)	26 (33.3)	5 (31.3)	80 (35.9)		56 (35.4)	19 (42.2)	75 (36.9)	
	Negative	13 (10.8)	0 (0)	9 (11.5)	0 (0)	22 (9.9)		4 (2.5)	17 (37.8)[b]	21 (103)	
sabA	On	64 (53.3)	4 (44.4)	39 (50.0)	9 (56.3)	116 (52.0)	0.950	67 (42.4)	40 (88.9)[b]	107 (52.7)	< 0.001
	Off	43 (35.8)	3 (33.3)	27 (34.6)	5 (31.3)	78 (35.0)		63 (39.9)	5 (11.1)[b]	68 (33.5)	
	Negative	13 (10.8)	2 (22.2)	12 (15.4)	2 (12.5)	29 (13.0)		28 (17.7)	0 (0)[b]	28 (13.8)	
sabB	On	23 (19.2)	3 (33.3)[b]	27 (34.6)[b]	8 (50.0)[b]	61 (27.4)	0.003	59 (37.3)	0 (0)[b]	59 (29.1)	< 0.001
	Off	35 (29.2)	4 (44.4)	28 (35.9)	6 (37.5)	73 (32.7)		72 (45.6)	0 (0)[b]	72 (35.5)	
	Negative	62 (51.7)	2 (22.2)[b]	23 (29.5)[b]	2 (12.5)[b]	89 (39.9)		27 (17.1)	45 (100)[b]	72 (35.5)	
hopZ	On	43 (35.8)	3 (33.3)	28 (35.9)	4 (25.0)	78 (35.0)	0.845	56 (35.4)	17 (37.8)	73 (36.0)	0.773
	Off	77 (64.2)	6 (66.7)	50 (64.1)	12 (75.0)	145 (65.0)		102 (64.6)	28 (62.2)	130 (64.0)	
**Total**		120 (100)	9 (100)	78 (100)	16 (100)	223 (100)		158 (100)	45 (100)	203 (100)	

[a]Global *p* values between disease groups or subgroups were assessed by Chi-Square test or Fisher Exact test. [b]Adjusted $p < 0.05$ versus NAG group or Sg1 was determined by Fisher's exact test (Bonferroni method). Sg1 = subgroup 1, Sg2 = subgroup 2, OMP = outer membrane protein, NAG = non-atrophic gastritis, PUD = peptic ulcer disease, AG/IM = atrophic gastritis / intestinal metaplasia, GC = gastric cancer, Sg1 = subgroup 1, Sg2 = subgroup 2.

Table S8. Distribution of T4SS components in *H. pylori* subgroups.

T4SS	Genotypes		Sg1 (*n*=158)	Sg2 (*n*=45)	Total (*n*=203)	P^a
*cag*PAI	Presence[§]	Y	153 (96.8%)	1 (2.2%)	154 (75.9%)	<0.001
		N	5 (3.2%)	44 (97.8%)	49 (24.1%)	
	Completeness[§§]	Y	151 (95.6%)	0	151 (74.4%)	<0.001
		N	2 (1.3%)	1 (2.2%)	3 (1.5%)	
	cagA presence	Y	152 (96.2%)	0	152 (74.9%)	<0.001
		N	6 (3.8%)	45 (100%)	51 (25.1%)	
comB	Presence[§] and completeness[§]	Y	158 (100%)	45 (100%)	203 (100%)	-
		N	0	0	0	
tfs3	Presence[§]	Y	126 (79.7%)	17 (37.8%)	143 (70.4%)	<0.001
		N	32 (20.3%)	28 (62.2%)	60 (29.6%)	
	Completeness[§§]	Y	18 (11.4%)	10 (22.2%)	28 (13.8%)	<0.001
		N	108 (68.4%)	7 (15.6%)	115 (56.7%)	
	ctkA	Y	79 (50.0%)	2 (4.4%)	81 (39.9%)	<0.001
		N	79 (50.0%)	43 (95.6%)	122 (60.1%)	
tfs4	Presence[§]	Y	148 (93.7%)	34 (75.6%)	182 (89.7%)	0.001
		N	10 (6.3%)	11 (24.4%)	21 (10.3%)	
	Completeness[§§]	Y	20 (12.7%)	26 (57.8%)	46 (22.7%)	<0.001
		N	128 (81.0%)	8 (17.8%)	136 (67.0%)	
	dupA	Y	18 (11.4%)	23 (51.1%)	41 (20.2%)	<0.001
		N	140 (88.6%)	22 (48.9%)	162 (79.8%)	
	Type	*tfs4a*[b]	3 (1.9%)	5 (11.1%)	8 (3.9%)	<0.001
		tfs4b[b]	14 (8.9%)	18 (40.0%)	32 (15.8%)	
		t*fs4c*	2 (1.3%)	2 (4.4%)	4 (2.0%)	
		tfs4-other[§§§b]	129 (81.6%)	9 (20.0%)	138 (68.0%)	
Combinations	*cag*PAI+*tfs3*		121 (79.1%)	1 (100%)	122 (79.2%)	0.607
	*cag*PAI+*tfs4*		143 (93.5%)	1 (100%)	144 (93.5%)	0.791
	tfs3+*tfs4*		118 (93.7%)	15 (88.2%)	133 (93.0%)	0.411
	tfs3+*tfs4a*[b]		1 (0.8%)	1 (5.9%)	2 (1.4%)	0.001
	tfs3+*tfs4b*[b]		14 (11.1%)	7 (41.2%)	21 (14.7%)	
	tfs3+*tfs4c*		2 (1.6%)	1 (5.9%)	3 (2.1%)	
	All T4SS[b]		113 (71.5%)	1 (2.2%)	114 (56.2%)	<0.001

[§]The presence of the T4SS was established by the assessment of the existence of either one of the following genes: *xerT, virB2, virB3, virB4, virB6, virB7, virB8, virB9, virB10, virB11, virD2, virD4*, and *topA*. For *cag*PAI presence was established by the assessment of the existence of either one of the following genes: *virB1, virB2, virB3, virB4, virB6, virB7, virB8, virB9, virB10, virB11,* and *virD4*. [§§]The presence of all *virB2, virB3, virB4, virB6*, (in the case of *cag*PAI, *virB7* is included), *virB8, virB9, virB10, virB11, virD2*, and *virD4* genes were used to determine the level of completeness of each T4SS. [§§§]unclassified and incomplete *tfs4* type. [a]Global *p* value between subgroups was assessed by Fisher's exact test or Chi square test. [b]Adjusted $p < 0.05$ versus subgroups was determined by Fisher's exact test (Bonferroni method). Y: yes, N: no. The global p value between disease groups were assessed by Chi-Square test. Sg1 = subgroup 1, Sg2 = subgroup 2

Figure S2. (A) Pairwise correlations among the presence of virulence determinants were assessed using Pearson's correlation coefficient, and the results were adjusted for multiple testing using the Bonferroni method. Red color indicated the positive, blue color indicated negative correlation between the variables. **(B)** Graphic representation of significant virulence determinants linked to gastric diseases in *H. pylori*-Mon strains by use of heatmap.

A

	cagA presence	*sabB presence*	*frpB2 presence*	*hopD presence*	*ceuE presence*	Complete *tfs4 presence*	*tfs4b* type presence	*vacA* types (s1i1m1 presence)	*hopQ* types (I presence)	*oipA* "on"	*sabB* "on"
cagA present	1										
sabB present	0.62	1									
frpB2 present	0.822	0.589	1								
hopD present	-0.303	-0.511	-0.324	1							
ceuE present	-0.461	-0.544	-0.476	0.324	1						
Complete *tfs4 present*	-0.39	-0.403	-0.299	0.184	0.387	1					
tfs4b type present	-0.323	-0.32	-0.205	0.141	0.304	0.819	1				
vacA types (s1i1m1 present)	0.603	0.746	0.556	-0.44	-0.536	-0.362	-0.315	1			
hopQ types (I present)	0.815	0.67	0.768	-0.34	-0.519	-0.345	-0.293	0.666	1		
oipA "on"	0.899	0.589	0.811	-0.299	-0.452	-0.348	-0.26	0.556	0.768	1	
sabB "on"	0.342	0.5	0.317	-0.307	-0.292	-0.196	-0.158	0.467	0.331	0.317	1

Black colored correlation r: p < 0.05
Pink colored correlation r: p > 0.05

B

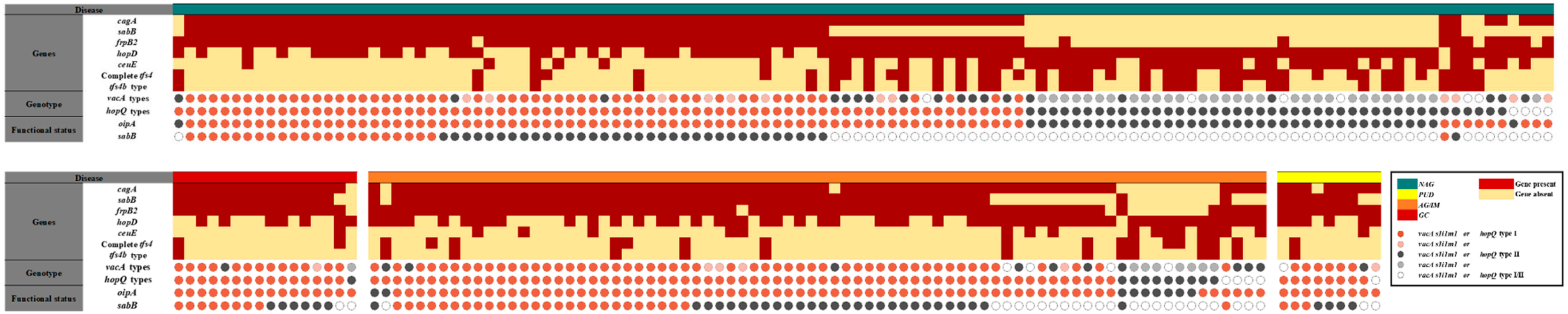

Figure S3. The GWAS Q-Q plot matched the importance of the SNPs and k-mers to the respective plots. The dot represents a SNP (A), and k-mer (B). Both axis represent *p* – significance level.

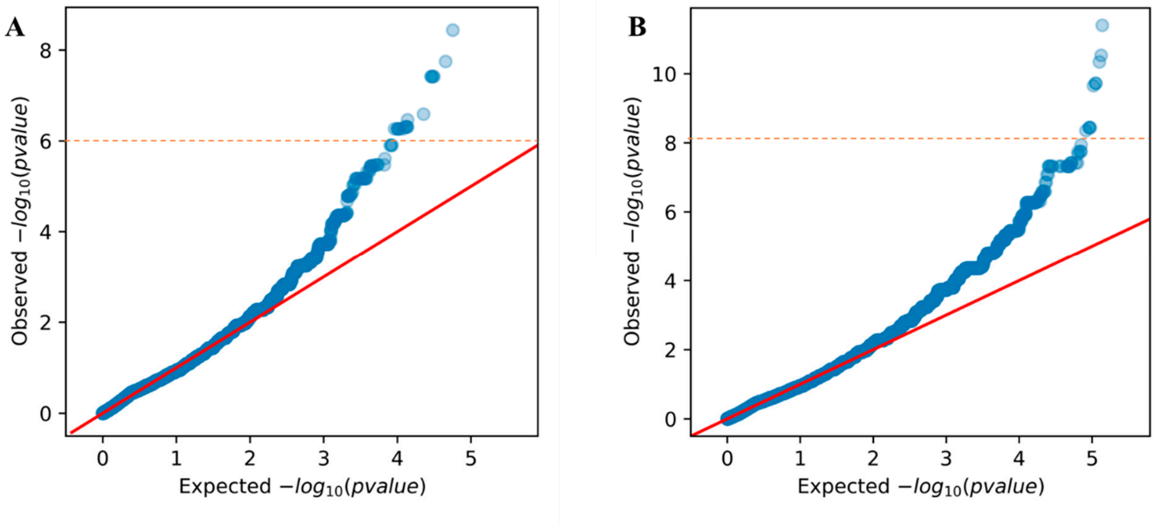

Table S9. Significant synonymous SNPs and kmers for GC were determined in genome-wide association studies of 158 *H. pylori* strains from Sg1.

Types	*P*	Gene locus in 26695	Gene name	Position in the genome	Position in the gene	Safe genotype	Risk genotype	Frequency of Non-GC/GC, %	Predicted function
SNP	$3.80x10^{-9}$	HP0502	*hpyAXII*	531001	765	A	G	3.5/40.0	R-M system, adenine methyltransferase
SNP	$3.44x10^{-7}$	HP0463	hyp[a]	482078	705	G	A	6.3/46.7	R-M system type I, SAM-dependent DNA methyltransferase
SNP	$5.39x10^{-7}$	HP0465	hyp[a]	487322	564	A	G	2.8/33.3	motility associated factor glycosyltransferase family protein
SNP	$5.39x10^{-7}$	HP0967	*vapD*	1026586	147	G	A	2.8/33.3	endoribonuclease
k-mer	$4.49x10^{-11}$	HP0193	*frdC*	200376-200412	366-402	other[b]	AAACCCGGTGAGG GCTTGAATAAACCA CAAACTCGTA	8.4/66.7	fumarate reductase cytochrome b-556 subunit
k-mer	$1.87x10^{-10}$	HP0690	*fadA*	741654	1095	G	A	2.1/40.0	acetyl-CoA acetyltransferase
k-mer	$3.62x10^{-9}$	HP1294	*rpsD*	1369068-1369071	300-303	GGAT	AGAC	2.8/40.0	30S ribosomal protein S4
k-mer	$3.80x10^{-8}$	HP0609	*faaA*	655274	8017	C	T	2.1/40.0	vacuolating cytotoxin domain-containing protein
k-mer	$3.80x10^{-8}$	HP0502	hyp[a]	530972-531032	735-795	other[b]	TTTTTTAAAAGATA AAAGAGTGCGTTT GGAGTATTTTAAAA AATTGTTTCAAGGA CACCCT	2.1/40.0	adenine methyltransferase

*Hypothetical protein gene. [b]The other motifs than risk genotype-motif considered as safe genotype.
